# Serological assessment of collagen fragments and tumor fibrosis may guide immune checkpoint inhibitor therapy

**DOI:** 10.1186/s13046-021-02133-z

**Published:** 2021-10-16

**Authors:** Christina Jensen, Neel I. Nissen, Claus S. Von Arenstorff, Morten A. Karsdal, Nicholas Willumsen

**Affiliations:** Biomarkers & Research, Nordic Bioscience, 2730 Herlev, Denmark

**Keywords:** Biomarker, Immune checkpoint inhibitor, Immunotherapy, T cells, Tumor fibrosis, Fibroblasts, Collagen, Extracellular matrix

## Abstract

Despite the overall clinical success of immune checkpoint inhibitors (ICIs) for treating patients with solid tumors, a large number of patients do not benefit from this approach. Consequently, there is a need for predictive biomarkers. The most prevalent biomarkers such as PD-L1 expression and tumor mutational burden (TMB) do not reliably predict response to ICIs across different solid tumor types suggesting that a broader view of regulating factors in the tumor microenvironment is needed. Emerging evidence indicates that one central common denominator of resistance to ICIs may be fibrotic activity characterized by extracellular matrix (ECM) and collagen production by cancer-associated fibroblasts (CAFs). A fibroblast-and collagen-rich stroma attenuates immunotherapy response by contributing to inhibition and exclusion of T cells. Here we review opportunities and limitations in the utilization of the most prevalent biomarkers for ICIs and elaborate on the unique opportunities with biomarkers originating from the activated fibroblasts producing an impermeable ECM. We propose that ECM and collagen biomarkers measured non-invasively may be a novel and practical approach to optimize treatment strategies and improve patient selection for ICI therapy.

## Background

Immunotherapy with immune checkpoint inhibitors (ICIs) has revolutionized the treatment and outcome of patients with several solid tumor types [1, 2]. Since the first FDA approval of anti-cytotoxic T lymphocyte antigen 4 (CTLA-4) for treatment of metastatic melanoma, a range of anti-programmed cell death protein 1 (PD-1) and anti-PD1 ligand 1 (PD-L1) therapies have been FDA approved for a large number of cancer types, including melanoma, non-small cell lung cancer (NSCLC), kidney cancer, and liver cancer [3]. Currently, long-term responses (2-3-year survival rate) are only seen in 30-60% of patients [4, 5]. The search for common denominators, across solid tumor types to identify non-responders is essential to advance cancer treatments.

Improving our understanding of the complex mechanisms leading to resistance and anti-tumor immunity is crucial for discovering promising therapeutic targets and identifying novel potential biomarker strategies for cancer immunotherapy [6]. There is an unmet medical need for practical and predictive biomarkers that can guide patient selection and therapy decisions to increase clinical outcome, avoid adverse events in patients not likely to respond to a given treatment, and limit the high costs related to treating non-responders with expensive medication [7].

The complex and dynamic tumor microenvironment that varies between individual patients and tumor types challenges the validation of biomarkers, and no biomarker assays have yet shown consistent and robust predictive value for ICI therapy across different solid tumor types, possibly because no common denominator has been identified [8].

In an attempt to categorize and profile patients in the context of immuno-oncology, Chen et al. and others have tried to define the variety of cellular and extracellular components of the tumor microenvironment as three broad immune profiles that can predict response to immunotherapy; the immune-inflamed tumor type, the immune-excluded tumor type, and the immune-desert tumor type [6, 9]. The immune-inflamed tumor type is characterized by high levels of T cells in proximity to tumor cells and associated with efficient ICI response [9, 10]. The immune-desert tumor type is characterized by few T cells in the tumor microenvironment while the immune-excluded tumor type is characterized by T cells that are excluded from the tumor and instead trapped in a fibroblast-and collagen-rich stroma [11]. Both immune-excluded and desert phenotypes are associated with poor response to ICI treatment [9]. Interestingly, several recent studies suggest that extracellular matrix (ECM) proteins, collagens, cancer-associated fibroblasts (CAFs), transforming growth factor-beta (TGF-β), and tumor fibrosis have key roles in resistance to immunotherapy [11–16]. These stromal components are found to inhibit the T cell infiltration and T cell activity, which are crucial for clinical response to anti-PD-1/PD-L1 therapy [10–22]. The reasons for response and resistance are diverse and other stromal components of the tumor microenvironment such as myeloid lineage cells, natural killer (NK) cells, B cells, and the vasculature may also positively or negatively influence the ability of ICIs to induce an effective anti-tumor immune response [9, 23–25]. However, one central common denominator of resistance to ICIs across different solid tumor types may be the ECM produced by CAFs, and the fibrotic reaction, limiting access of immune cells. This dense ECM matrix, of which collagens are the main components, is the limiting factor for organ function, in liver, lung, kidney, and heart fibrosis [26].

This review aims to discuss opportunities and limitations in the utilization of the most prevalent biomarkers for ICI therapy and elaborates on the unique opportunities with serological collagen biomarkers originating from activated fibroblasts, alone, and in combination, within the context of cancer immunotherapy.

### Opportunities and limitations with the most prevalent biomarkers for ICI therapy

The current immuno-oncology biomarker landscape primarily covers assessment of tumor antigens (high levels of microsatellite instability (MSI-H)/deficient mismatch repair (dMMR), expression of neoantigens, tumor mutational burden (TMB)), inflamed tumor markers (inflammation gene signatures, tumor-infiltrating lymphocytes (TILs)), immune suppression markers (PD-L1, LAG-3, myeloid-derived-suppressor cells (MDSCs), tumor-associated macrophages (TAMs), regulatory T cells (Tregs)) and host microenvironmental factors (microbiome) (Table 1) [9, 20, 23, 27–32]. Common for all these biomarkers is an association with the activation of T cells, which are crucial for response to immunotherapy. However, though the T cells are activated, the anti-tumor immune response can still be blocked by the retention of T cells in the dense collagen stroma that surrounds the tumor (i.e. the immune-excluded tumor type) [9]. While exploratory research is ongoing to try to better characterize and define common denominators of an immune-excluded phenotype [33], currently, there are no clinically applicable biomarkers that can identify the patients with the immune-excluded phenotype that despite activated T cells, do not respond to immunotherapy.

**Table 1:** Selected factors/biomarkers of response and resistance to ICIs

Response	Resistance
Tumor mutational burden (TMB)	Myeloid-derived-suppressor cells (MDSCs)
Neoantigen expression	Tumor-associated macrophages (TAMs)
High levels of microsatellite instability (MSI-H)	Regulatory T cells (Tregs)
Deficient mismatch repair (dMMR)	Extracellular matrix (ECM)
Tumor-infiltrating lymphocytes	Collagen
Inflammation gene signature	Cancer-associated fibroblasts (CAFs)
PD-L1 expression	Transforming growth factor-beta (TGF-β)
LAG-3 expression	
Host factors (Microbiome)	

It has been suggested that successful biomarkers depend on three features: (a) a biological role related to tumor development and progression; (b) detectability with robust, reliable, and clinically applicable assays; and (c) a prognostic or predictive value that is validated in clinical trials [34].

Blood-based biomarkers are attractive, due to the ease of drawing a blood sample compared to tumor tissue, and because it is associated with minimal pain for the patients [35]. Blood-based biomarkers can give an overall molecular status of the patient, which may overcome intratumor heterogeneity. Another advantage of blood-based biomarkers is the possibility of frequent testing during patient follow-up, compared to tumor tissue biopsies that often cannot be performed repeatedly. As technical, analytic, and study parameters may influence the measurement and interpretation of blood-based protein biomarkers, assessment of reproducibility, sample acquisition, freeze-thaw cycles, storage conditions, and assay parameters are important for reliable biomarker results [36]. The clinical applicability of blood-based biomarkers should be well validated in large studies where confounding factors such as age, gender, body mass index, food intake, co-morbidities, and prior treatments are taking into account [37].

The two most investigated biomarkers in the immuno-oncology space are PD-L1 expression and TMB and while these have shown promising results in some solid tumor types they also contain technical and biological limitations [20, 30–32].

#### PD-L1 expression

Detection of PD-L1 tumor tissue expression with immunohistochemistry emerged as one of the first and most studied biomarkers for anti-PD-1/PD-L1 therapy based on the assumption that PD-L1 should be expressed for anti-PD-1/PD-L1 to induce a response [38]. But, across different solid tumor types treated with anti-PD-1/PD-L1 drugs, PD-L1 has only been shown to be predictive in around 30% of cases [30]. PD-L1 has been FDA approved as a biomarker for use in NSCLC, head and neck squamous cell carcinoma, bladder, breast, cervical and gastric cancer, but though extensively studied in melanoma, it has not been FDA approved for this indication [29, 30]. When assessing the PD-L1 biomarker in several melanoma studies, a correlation between PD-L1 expression and response to anti-PD-1 therapy was only seen in five out of eight studies [20]. One of the main concerns is that a subset of patients that have low PD-L1 levels will respond to anti-PD-1 therapy.

There are a variety of limitations concerning PD-L1 expression measured in a tissue biopsy including differences in PD-L1 thresholds, differences in diagnostic antibodies applied, different detection assays, the heterogenicity of PD-L1 expression between serial sections of a primary tumor and metastatic sites, the dynamic PD-L1 expression over time, and that it is a semiquantitative approach based on visual assessments [29, 39].

Although PD-L1 expression is the most investigated and best-validated biomarker of response to ICI therapy, at least more refinement, optimization, and validation are needed for PD-L1 expression to be a useful and reliable predictive biomarker [29, 40].

#### Tumor mutational burden (TMB)

Modern high-throughput technologies including mass spectrometry, whole-exome, and RNA sequencing allow comprehensive profiling of individual patients and assessment of thousands of genes and proteins [41, 42]. High TMB, which may induce more tumor-specific neoantigens that can enhance T cell responses against tumors, has been widely investigated as a predictive biomarker for ICIs in solid tumor types such as melanoma, breast cancer, renal cancer, and NSCLC [20, 28, 31, 32, 43]. Recently, Litchfield et al. have assessed whole-exome and transcriptomic data for > 1000 ICI-treated patients across seven tumor types and shown that clonal TMB is a strong predictor of response followed by TMB and CXCL9 expression [44]. Moreover, based on the successful phase 2 KEYNOTE-158 study, the FDA has granted accelerated approval of pembrolizumab (anti-PD-1) for the treatment of patients with unresectable or metastatic TMB-high solid tumors whose cancer has progressed after previous therapy [45]. However, a new TCGA analysis with over 10,000 patient tumors failed to support the application of TMB-high as a biomarker for ICI therapy in all solid tumor types and suggests that further tumor-specific studies are needed [46].

Despite encouraging findings of TMB in some tumor types, such as NSCLC [28, 47], and advantages with high-throughput technologies, the broad applicability of TMB as a biomarker across all solid tumors is unclear. It is challenging to detect the exact neoantigens that induce T cell responses, and TMB assessment in a tissue biopsy is limited by patient surgical risk, intratumor heterogeneity, different cut-off values, detection methods, and overlap between responding and non-responding patients [20, 31, 32, 48, 49].

#### Liquid biopsy-based biomarkers

In the pursuit of overcoming the tumor heterogeneity detected in tumor biopsies, and the fact that tumor biopsies are challenging and sometimes impossible to obtain [35], assessment of circulating biomarkers originating from tumors including circulating tumor cells, proteins, tumor DNA, and tumor RNA have been investigated as biomarkers [50]. A decrease in circulating tumor DNA (ctDNA) assessed in peripheral blood during ICI therapy has been found to correlate with response to combined anti-CTLA-4 and anti-PD-1 therapy in metastatic melanoma patients [51]. Exosomal PD-L1 from tumors has been identified as an alternative mechanism of PD-L1 activity, which suppresses T cell activation suggesting that exosomal PD-L1 represents a potential therapeutic target and novel predictive biomarker for anti-PD-1 therapy [52, 53]. Low soluble PD-L1 plasma levels have also been discovered as a potential biomarker for the prediction of response to anti-PD-1 therapy in NSCLC patients [54].

However, similar to PD-L1 expression assessed with immunohistochemistry, the clinical application of these tumor-derived liquid biopsies is also challenged by limited standardized methods, discordant results, and false negatives [50].

The biomarkers reviewed in this section, such as PD-L1 expression, TMB, and ctDNA have some technical limitations. Moreover, though the biomarkers are related to T cell activity, their predictive value for ICI response varies across different solid tumor types suggesting that a broader view of regulating factors in the tumor microenvironment is needed.

### ECM biomarkers, with non-invasive collagen biomarkers as an example, for personalized healthcare in immuno-oncology – current evidence

#### Tumor microenvironment and the role of collagens in immuno-oncology

Increasing evidence suggests that the ECM, the non-cellular component of the tumor microenvironment, influences response to immunotherapy [33, 55]. The ECM is composed of the basement membrane and the interstitial matrix. The basement membrane is a sheet-like ECM layer located beneath the endothelial and epithelial cells, serving as a barrier to the underlying stroma, and is primary composed of type IV and VIII collagen, laminin, nidogen, and perlecan [56]. The interstitial matrix is responsible for tissue structure and is mainly composed of type I, III, V, VI, and XI collagen, which are primarily produced by fibroblasts [57].

Collagens are the most abundant ECM proteins in the tumor microenvironment across different solid tumor types, and in addition to their well-known role in tumor progression and metastasis, several recent studies suggest that collagens have a direct role in resistance to ICI therapy [11–16, 58–60]. It has been shown that an immune-excluded tumor phenotype is characteristic of having CAF activity, and a collagen-rich stroma (tumor fibrosis) that influences the efficacy of ICIs by acting as a protective shield for the tumor by trapping the T cells [11, 16, 61, 62]. CAFs are one of the most abundant cell types in the tumor microenvironment, and in addition to being the main producer of fibrotic ECM proteins and collagens, CAFs have multiple immunosuppressive functions and secrete numerous chemokines, cytokines, and proteases, such as TGF-β, VEGF, CXCL12/CXCR4, interleukin-6, and matrix metalloproteinases (MMPs) [58, 63]. In metastatic urothelial cancer patients, lack of response to anti-PD-L1/PD-1 therapy was associated with TGF-β signaling in fibroblasts and CD8+ T cell excluded tumors, where T cells were retained in a fibroblast-and collagen-rich peritumoral stroma [11, 12]. Moreover, co-administration of anti-PD-L1 and anti-TGF-β antibodies in both a mammary carcinoma mouse model and in colon carcinoma mouse models have been shown to induce T cell infiltration into the tumor, which was associated with an anti-tumor response [11, 64, 65]. The association between upregulated ECM and TGF-β genes, and T cell excluded tumors with a peri-tumoral location of CD8+ T cells has also been detected in hepatocellular carcinoma patients supporting that tumor fibrosis affects the anti-tumor immune response across different tumor types [16]. Moreover, a large pan-cancer analysis has shown that an ‘ECM-up’ signature is activated by TGF-β signaling in CAFs and that it is the ‘ECM-up’ signature per se that is associated with anti-PD-1 resistance, and not just CAFs or TGF-β activation [13]. Interestingly, the predictive performance of the ‘ECM-up’ signature outperformed a T cell inflamed signature and mutational load alone [13]. TGF-β has a highly pleiotropic nature with both immune regulatory mechanisms and a critical role in generating the fibrotic tumor microenvironment [66]. TGF-β family members including TGF-β1 signal through TGF-βR to promote the expression of ECM proteins including collagen, fibronectin, tenascin C, and laminin [67, 68]. Cancer cells secrete TGF-β that promotes CAF contractility and secretion of TGF-β and MMPs leading to collagen production and degradation, respectively [69]. TGF-β signals by activating non-canonical signaling pathways such as RhoA/ROCK, and by activating canonical signaling pathways to induce SNAIL1 and TWIST1 gene transcription [66, 70–72].

Tumor fibrosis and collagens also have direct immunosuppressive functions. A high-density collagen matrix induces the downregulation of cytotoxic activity markers and upregulation of regulatory T cell markers [15]. Increased collagen levels correlate with an increased amount of exhausted CD8+ T cells and resistance to PD-1/PD-L1 blockade in lung tumors [14]. This CD8+ T cell exhaustion is induced by collagen binding to the collagen receptor leukocyte-associated immunoglobulin-like receptor 1 (LAIR-1), which suppresses T cell activity through SHP-1 signaling supporting a direct immune regulating role of collagen [14]. A recent study also shows the relevance of blocking the LAIR-1-collagen interaction as a novel checkpoint inhibitor approach in cancer [73]. Cleavage of collagen and ECM proteins by MMPs and other remodeling enzymes generate a variety of bioactive peptide fragments that may have signaling properties and act as chemokines, cytokines, or interact with immune regulators such as LAIR-1 [25, 74].

**Fig. 1:**
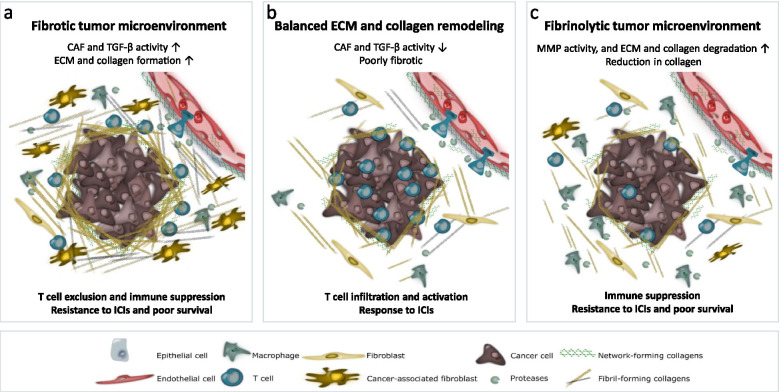
Response to immunotherapy is associated with balanced collagen formation and degradation in the tumor microenvironment. **a** A fibrotic tumor microenvironment characterized by high cancer-associated fibroblast (CAF) activity, transforming growth factor-beta (TGF-β) signaling, and extracellular matrix (ECM) and collagen formation is associated with T cell exclusion, immune suppression, and poor response to immune checkpoint inhibitors (ICIs). **b** T cell immunity and response to ICIs are associated with a balanced degree of ECM/collagen formation and degradation, and less CAF and TGF-β activity. **c** A fibrinolytic tumor microenvironment characterized by high matrix metalloproteinase (MMP) activity, and ECM and collagen degradation is associated with immune suppression and resistance to ICIs

Together, the studies highlighted in this section reveal the central roles of upregulated TGF-β-signaling in CAFs and the increased production of collagens in inducing immune evasion, immune exclusion, and resistance to ICIs. In contrast, it has also been shown that depletion of fibroblasts and reduction of collagens/tumor fibrosis can lead to immune suppression and poor survival [75, 76]. Chen et al. have recently shown in a mouse model with spontaneous pancreatic cancer that a significant reduction in total stromal type I collagen can accelerate pancreatic tumor progression and augment suppression of CD8+ T cells leading to decreased overall survival [76]. This proposes that optimal T cell immunity and response to immunotherapy are associated with a balanced degree of ECM/collagen formation and degradation and that both excessive collagen production and excessive collagen degradation associate with resistance and poor survival outcomes (Fig. 1). Together, this highlights a need and a potential for collagen-based biomarkers that assess the activity of TGF-β, CAFs, collagen formation, and collagen degradation.

#### Limitations of assessing tumor fibrosis and collagens in tumor tissue biopsies - activity versus status

The findings that collagens seem to have a direct role in resistance to ICI therapy are mainly based on collagen mRNA expression and trichrome staining for total collagen in tissue biopsies [11–14, 16]. Such tissue-based measures only provide a snapshot of the status of the total collagen content which makes it difficult to distinguish increased collagen deposition and degradation. Moreover, it is difficult to monitor patients as serial biopsies are rarely an option. Tumor fibrosis is an active process that includes the activation of fibroblasts and CAFs, the release of TGF-β, the production of collagen, and degradation of collagens by proteases such as MMPs [59]. Serological assessment of collagen fragments and fibrotic activity may be ideally suited as a personalized health care tool that can provide additional information to the static measure of tumor fibrosis assessed by tissue-based technologies such as immunohistochemistry or similar.

#### The potential of blood-based collagen biomarkers in immuno-oncology

**Fig. 2:**
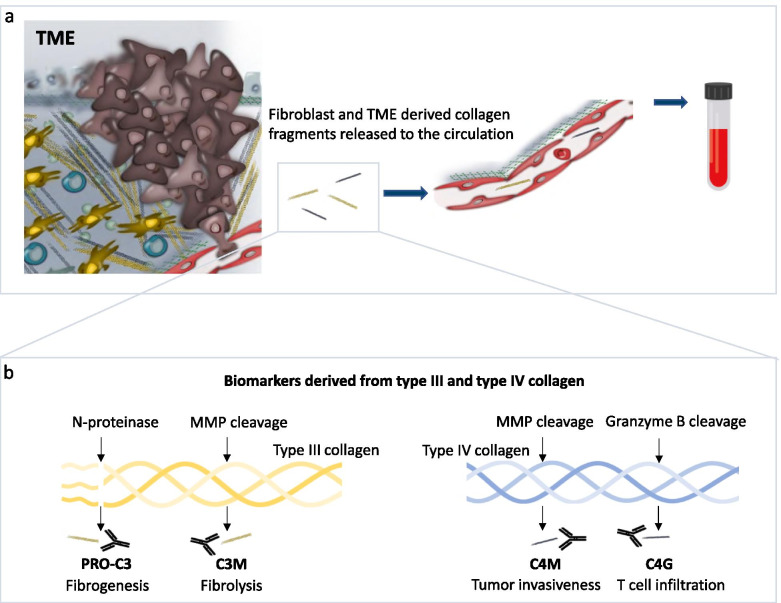
Fibroblast and tumor microenvironment-derived collagen fragments as blood-based biomarkers. **a** Consequent to increased fibroblast activity and protease-mediated collagen remodeling in the tumor microenvironment (TME), specific protein fragments are released into the circulation and can be used as non-invasive biomarkers assessed in a liquid biopsy (serum or plasma). Modified from Nissen et al., J Exp Clin Cancer Res, 2019. **b** The neo-epitope biomarker technology is based on monoclonal antibodies, which enables assessment of remodeling of specific collagens with diverse proteases. Measurement of the pro-peptide of type III collagen (PRO-C3) reflects fibrogenesis, while MMP degraded type III collagen (C3M) reflects fibrolysis. MMP degradation of the main basement membrane protein type IV collagen (C4M) reflects tumor invasiveness while granzyme B degraded type IV collagen (C4G) reflects T cell infiltration

Focusing on specific subdomains of proteins, such as the collagen pro-peptides, rather than quantification of the total proteins with standard assay technologies, may provide an advantage. Collagen contains pro-peptides which are released during collagen synthesis. These pro-peptides are thus an indirect measure of CAF activity, collagen production, and fibrogenesis [57, 77]. In direct alignment, quantification of special degradation epitopes, provides a completely different type of information, on tissue degradation by specific cell types [78]. Par example, quantification of neo-epitopes which are the product of a signature ECM protein and a specific protease makes it possible to assess separate processes such as MMP-mediated collagen degradation and collagen formation of specific collagens [79, 80]. During tissue remodeling, collagens are remodeled and these specific protein fragments are released into the circulation and can be used as non-invasive biomarkers assessed in a liquid biopsy (serum or plasma) by quantitative immunoassays compared to a semi-quantitative approach such as trichrome staining of tissue (Fig. 2) [79, 80].

Two examples of neo-epitope assays are related to the two most abundant collagens in the ECM, type III (interstitial matrix) and type IV collagen (basement membrane) that have different positions and roles in the tumor microenvironment (Fig. 2) [59]. The PRO-C3 biomarker measures the pro-peptide released during type III collagen formation, which makes it feasible to assess the production and deposition of type III collagen [81], whereas type III collagen degraded by MMP can be quantified by the C3M biomarker [82]. PRO-C3 is found to be released by TGF-β stimulated CAFs in vitro [83], and high PRO-C3 in pre-treatment serum has been shown to predict poor overall survival in metastatic melanoma patients treated with anti-CTLA-4 or anti-PD-1 therapy [83–85]. These studies suggest that PRO-C3 can be used to assess CAF activity and the deposition of the collagen-rich peritumoral stroma that is associated with resistance to ICI therapy [11–16].

In the pursuit of identifying patients with high T cell infiltration, a biomarker measuring granzyme B degraded type IV collagen products (C4G) in serum has been developed [86]. The discovery of C4G was based on a study from Prakash et al. showing that granzyme B promotes cytotoxic lymphocyte transmigration over the basement membrane by degrading components such as type IV collagen [87]. High levels of this C4G biomarker at baseline could identify melanoma patients responding to anti-CTLA-4 treatment and when combined with low PRO-C3, this biomarker combination could identify additional responding patients [86]. Another study shows in vivo how C4G levels are increased in serum after inducing T cell activity with antibodies that block the LAIR-1-collagen interaction [73]. Here, the increase in C4G was observed at the time of tumor eradication supporting that the fragments were derived from the tumor microenvironment as a result of T cell activation and effector function. Interestingly, while the granzyme B degraded type IV collagen (C4G) fragments were increased in melanoma patients responding to anti-CTLA-4 treatment and modulated by inducing T cell activity in vivo, another MMP-degraded type IV collagen (C4M) fragment showed the opposite prognostic potential in the melanoma patients treated with anti-CTLA-4 and was not modulated by inducing T cell activity in vivo [73, 86]. This suggests that different protease-generated neo-epitopes on the same collagen reflect different biological processes (Fig. 2) [73, 84, 86]. Furthermore, these studies support the value of measuring specific neo-epitopes and not just the total protein.

The ECM is highly dynamic and constantly remodeled, and with these neo-epitope activity biomarkers, it is possible to assess processes such as fibrotic activity and T cell activity. Interestingly, it has been shown that high type III collagen turnover (C3M/PRO-C3 ratio) measured in the circulation is superior to hyaluronan assessed in a tissue biopsy from matched pancreatic cancer patients in predicting response to a stromal modifier in combination with chemotherapy [88]. Despite concomitant overexpression of type III collagen and hyaluronan in pancreatic tumors, high type III collagen turnover (C3M/PRO-C3 ratio) has predictive value both in hyaluronan low and hyaluronan high patients suggesting that the tissue biopsy and liquid biopsy do not identify the same patients [88, 89]. This study highlights the unique value of measuring the fibrotic activity non-invasively compared to assessing the static fibrosis in a tumor biopsy, where it is difficult to distinguish fibrolysis and fibrogenesis.

### Future perspectives, and limitations, with non-invasive ECM biomarkers for ICI therapy

Non-invasive ECM biomarkers measure a systemic pool of the protein fragment of interest, either a formation or degradation epitope [80]. This type of biomarker quantification may overcome the intra-tumor heterogenicity that is a huge limitation when assessing PD-L1 expression and TMB in tumor tissue biopsies [90]. A tumor tissue biopsy only provides a snapshot of the status of the disease at a given time point in a smaller biopsy, which may be less representative whereas ECM biomarkers measured in the circulation reflect the entire fibrotic activity of the tumors.

Though these ECM biomarkers provide a unique value of non-invasive longitudinal monitoring that is not always possible with tumor tissue biopsies, tissue-based assessment for histological tumor diagnosis and staging at baseline will probably still be needed. Such a biopsy approach may also provide important complementary insight into ECM status in the tumor microenvironment e.g. T-cell trapping capacity [11]. One evaluation step could be to compare the non-invasive ECM biomarkers with the immune profiles; the immune-inflamed tumor type, the immune-excluded tumor type, and the immune-desert tumor type to examine if the non-invasive assessment of fibrotic activity correlates with the static fibrosis in the phenotypes or if the ECM biomarkers have additional potential.

As is observed with PD-L1 and TMB assessments, the non-invasive ECM biomarker levels are also overlapping between responding and non-responding patients. Considering the complex and dynamic nature of the tumor microenvironment, a single biomarker may not be sufficient, and a combination of biomarkers may likely be needed for optimal patient selection for immunotherapy [91, 92]. A combination of an initial tumor biopsy assessing PD-L1, TMB, and ECM status, and baseline and longitudinal assessment of ECM biomarkers in the circulation may have unique potential for patient phenotyping for ICIs. Non-invasive collagen biomarkers will most likely provide an additional value to tissue-based biomarkers such as PD-L1 expression and TMB because these collagen biomarkers reflect another important biology associated with resistance to ICIs compared to PD-L1 expression and TMB that are associated with T cell activity and response.

Though ECM biomarkers have an advantage over tissue-based biomarkers because they are measured in circulation, they may also have some limitations. As ECM remodeling is a physiological process taking place throughout the body, it must be evaluated if the ECM biomarkers derive from the tumor microenvironment. Because these ECM and collagen biomarkers are based on the assessment of specific post-translational modifications of a specific protein (neo-epitopes) compared to total protein, it reduces the systemic background of healthy collagen turnover and instead increases the specificity for pathological collagen turnover processes [79, 80]. The specific collagen turnover in the primary tumor and sites of secondary metastases is sufficiently high compared to in healthy tissue and benign and nonmalignant tissues of comparable organs (e.g. pancreatic cancer versus chronic pancreatitis, liver cancer versus cirrhosis, lung cancer versus idiopathic pulmonary fibrosis) [93–95]. Kehlet et al. showed that formation of type III collagen (PRO-C3), and MMP-degradation of type I, III, and IV collagen (C1M, C3M, and C4M) were elevated in stage IV CRC patients compared to stage I, II, and III CRC patients and healthy controls [96]. A similar pattern was seen for PRO-C3 in pancreatic cancer [95]. Furthermore, significantly elevated C3M levels have been detected in conditioned medium from CRC tumor core tissues cultured ex vivo, compared to conditioned medium from non-neoplastic adjacent tissues from the same patients [97]. Moreover, PRO-C3 was increased 5-fold in conditioned medium from CAFs as compared to normal fibroblasts [83].

The collagen turnover products have previously been shown to be slightly affected by age, in particular during menopause, suggesting that age should be accounted for when measuring ECM-related exploratory biomarkers in clinical studies [37]. Accordingly, when adjusting for age in the described immuno-oncology studies in this review, the prognostic potential of the ECM biomarkers remained significant supporting that the observed biomarker potential was not due to differences in age [84, 86]. Furthermore, as with all liquid biopsies, there are technical parameters such as sample acquisition, freeze-thaw cycles, measurement range, cut-off values, sensitivity, and specificity that should be considered [36].

Altogether, though limitations exist and additional validations should be performed, we think non-invasive ECM biomarkers can provide additive and unique value to the evolving landscape of biomarkers for ICI therapy.

## Conclusions

Although ICIs have revolutionized cancer treatment, durable responses are only seen in 30-60% of patients. In the pursuit of identifying biomarkers that predict therapy response, some of the most investigated markers such as PD-L1 expression and TMB have shown varying predictive value and have several technical and biological limitations. The reason why some patients do not respond, even though PD-L1 is present or they have high TMB, may be due to high fibrotic activity and a collagen-rich tumor that traps and inhibits the T cells. This collagen-rich area is known in other fibrotic diseases to limit organ function. Collagens are often an overlooked facet of immune regulation and tumor biology. By adding an additional layer of information, in terms of stratifying patients with collagen-rich/fibrotic tumors and patients without this thick collagenous matrix by measuring collagen fragments non-invasively in a simple blood sample, it is overwhelmingly likely that a subpopulation of “true responders” could be identified. As tumor fibrosis is a highly dynamic process, neo-epitope collagen biomarkers measured non-invasively may be a novel approach to optimize treatment strategies and improve patient selection for ICI therapy.

## Data Availability

Not applicable.

## Abbreviations

CAFs: Cancer-associated fibroblasts
ctDNA: Circulating tumor DNA
CTLA-4: Cytotoxic T lymphocyte antigen 4
dMMR: Deficient mismatch repair
ECM: Extracellular matrix
PRO-C3: Formation of type III collagen
C4G: Granzyme B degraded type IV collagen
ICI: Immune checkpoint inhibitors
LAIR-1: Leukocyte-associated immunoglobulin-like receptor 1
MMPs: Matrix metalloproteinases
C1M: MMP-degraded type I collagen
C3M: MMP-degraded type III collagen
C4M: MMP-degraded type IV collagen
MDSCs: Myeloid-derived-suppressor cells
MSI-H: High levels of microsatellite instability
NK: Natural killer
NSCLC: Non-small cell lung cancer
PD-1: Programmed cell death protein 1
PD-L1: PD1 ligand 1
Tregs: Regulatory T cells
TGF-β: Transforming growth factor-beta
TAMs: Tumor-associated macrophages
TILs: Tumor-infiltrating lymphocytes
TMB: Tumor mutational burden.
